# Acknowledgment to Reviewers of *Pharmaceuticals* in 2020

**DOI:** 10.3390/ph14020082

**Published:** 2021-01-22

**Authors:** Pharmaceuticals Editorial Office

**Affiliations:** MDPI AG, St. Alban-Anlage 66, 4052 Basel, Switzerland

Peer review is the driving force of journal development, and reviewers are gatekeepers who ensure that *Pharmaceuticals* maintains its standards for the high quality of its published papers. Thanks to the cooperation of our reviewers, in 2020, the median time to first decision was 14 days and the median time to publication was 32 days. The editors would like to express their sincere gratitude to the following reviewers for their precious time and dedication, regardless of whether the papers were finally published:
Abd El Hakim, YasminaLešnik, SamoAbdullah, AmmaraLevina, AvivaAbramson, Hanley N.Li, ChenshuangAbushouk, AbdelrahmanLi, FengzhiAdamo, RobertoLi, JingAdamson, David CoryLi, MengAdebisi, Adeola O.Li, MiaomiaoAdina, Turcu-StiolicaLi, Ruei-NianAdunyah, Samuel E.Li, TianhuAfantitis, AntreasLi, Wen-WuAfrin, SadiaLi, WenyiAgostino, MarkLi, Xiang-GuoAhamed, MuneerLiang, JunAhmed, SafeerLiao, Ai-HoAhn, JeonghyunLiesiene, JolantaAhn, TaehoLima, Camilo Henrique Da SilvaAime, SilvioLin, Cheng-WenAisa, Haji AkberLin, Hai-ShuAkhlaghi, Seyedeh ParinazLin, Hung-YinAl Rihani, SweilemLin, Jer-AnAlbert, SilviaLin, QishanAlberti, DiegoLin, WeifengAlbertini, MariaLinden, SaraAl-Dujaili, EmadLinder, BenediktAlexander, H. DenisLitak, JakubAlexandrova, ElenaLiu, HuolongAlford, ChrisLiu, RuiwuAlizargar, JavadLiu, YanhongAljabali, Alaa A. A.Liverani, ElisabettaAl-kinani, Ali AthabLlinares, José MiguelAllott, LouisLodola, AlessioAlonso-Moreno, CarlosLodyga-Chruscinska, ElżbietaAlsadeq, AmeeraLoeza Alcocer, Jose EmanuelAlvarez, AlejandraLok, Chun NamAlvarez, F. JavierLomberget, ThierryÁlvarez-Mercado, Ana I.Londono, BerlinAlves, C. HenriqueLopalco, AntonioAmadio, PatriziaLópez Lluch, GuillermoAMAROWICZ, RyszardLópez Nicolás, Jose ManuelAmmazzalorso, AlessandraLopez Rodilla, Jesus M.Amorós, AsunciónLópez, ÓscarAnas, Andrea Roxanne J.Łopusiewicz, ŁukaszAncuceanu, RobertLoureiro, Joana A.Andres, EmmanuelLovecká, PetraAndriolo, JessicaLow, MitchellAndriotis, Eleftherios G.Lozano-López, María VictoriaAngeli, AndreaLu, WenchaoAngelov, BorislavLuca, AndreiAngelovski, GoranLucas, Andrew T.Anisiewicz, ArturLucibello, MariaAnnibal, AndreaLudwiczuk, AgnieszkaAnsari, Mohammad YunusLudwig, Dale L.Antal, DianaŁukasiewicz, SylwiaAntal, IstvánLulla, ValeriaAntipova, VeronicaLuvisetto, SiroAntoni, GunnarLuwor, RodneyAraniciu, CătălinMacek Jílková, ZuzanaArino, JoaquinMach, MojmirArtalejo, Antonio R.Madala, Hanumantha RaoAsakura, HidesakuMadamsetty, Vijay SagarAshton, TrentMadka, VenkateshwarAso, YoshimasaMadoux, FranckAssaf, Khaleel IbrahimMaggi, DavideAstolfi, AndreaMaghsoodi, AmenehAudet-Walsh, ÉtienneMagri, GiuliaAuriemma, GiuliaMahli, AbdoAvgerinos, KonstantinosMahmood, IftekharAwatade, Nikhil TMainardi, MarcoAyabe, TatsuhiroMair, Lamar O.Azab, WalidMaisano, DomenicoAzer, Samy A.Maitra, RadhashreeBaek, In-HwanMaj, MalgorzataBagnoli, PaolaMajumder, PoulamiBai, RenrenMalapelle, UmbertoBaiocco, PaolaMalavasi, FabioBakashi, HamidMalejczyk, JacekBaker, MartinMalfa, Giuseppe AntonioBakowsky, UdoMalik, Anna R.Balalaeva, Irina V.Mallamaci, RosannaBalan, PrabhuMalouf, CamilleBaldassari, SaraMalpeli, GiorgioBaltina, LidiaMaltsev, Dmitrii S.Balzano, TizianoMalwal, Satish R.Bandea, ClaudiuMammadova-Bach, ElminaBano, GregorManca, Maria LetiziaBaqi, YounisMancinelli, RominaBarbalinardo, MariannaMandal, AbhirupBarbosa, Carlos MauricioMangiaterra, GianmarcoBarbosa, Raquel De MeloMangwandi, ChiranganoBarlocco, DanielaMannu, AlbertoBaron, ByronMansouri, KamelBartolini, ManuelaMånsson, SvenBartuzi, DamianManville, RianBarygin, Oleg I.Mao, XiaoboBasnet, PurusotamMarc, GabrielBatista, PatríciaMarceau, FrançoisBattistelli, CeciliaMarginǎ, DenisaBauer, EikeMarini, Herbert RyanBaykov, SergeyMarkowicz-Piasecka, MagdalenaBeck, Michael W.Marrazzo, AgostinoBeharry, Kay D.Marrocchi, AssuntaBehbahani, HomiraMartella, GiuseppinaBel’skaya, LyudmilaMartin Lawson, AtaBelaidi, Abdel AliMartínez Beamonte, RobertoBellmann-Sickert, KathrinMartinez Rodriguez, FlemingBellumori, MariaMarverti, GaetanoBelluzzi, ElisaMashima, RyuichiBeloukas, ApostolosMashruwala, AmeyaBeltowski, JerzyMaslov, Dmitry A.Benhar, ItaiMassalska, MagdalenaBergamo, PaoloMatarese, GiovanniBerger, GillesMatikonda, SiddharthBernabei, FedericoMattoscio, DomenicoBernardini, GiuliaMauceri, RodolfoBernardo, AntoniettaMauro, Adolfo GabrieleBeug, MichaelMavrogonatou, EleniBhandareh, Richie R.Mavromoustakos, ThomasBhatt, PriyankaMazel, VincentBhattacharjee, MrinalMazor, OhadBhattacharya, AbhisekMazzoni, MaraBianco, ArmandodorianoMcCarthy, ElaineBielenica, AnnaMcdonald, PeterBilewicz, AleksanderMcKenzie, Brienne A.Billamboz, MurielMcManus, KirkBilski, JanMeduri, AlessandroBinda, ElisaMeier, Robert J.Birner-Gruenberger, RuthMeissner, AnjaBIVONA, GiuliaMelas, MarilenaBjornstedt, MikaelMeléndez-Alafort, LauraBlack, DavidMelilli, Maria GraziaBlangetti, MarcoMelo, Sonia A.Blasiak, JanuszMelzig, MatthiasBoichuk, SergeiMendyk, AleksanderBolívar, JorgeMenendez, Jose CarlosBolla, Pradeep KumarMeng, GeBollinger, Justin L.Meo, Paolo LoBollong, MichaelMerlino, AntonelloBONAVIDA, BENJAMINMeseda, ClementBongiovanni, AlbertoMessore, AntonellaBonnet, Célia S.Mi, Fwu-LongBonnet, PascalMiastkowska, MałgorzataBono, NinaMiazek, ArkadiuszBoppana, Nithin B.Micale, NicolaBórawski, PiotrMichálek, MartinBorbone, NicolaMieczkowski, AdamBoroń, DariuszMihály, AndrásBorota, AnaMiki, YasuhiroBoschi, StefanoMikkilineni, LekhaBose, TanimaMikropoulos, ChristosBotana, Ana MaríaMilano, FrancescoBotez, ElisabetaMilavetz, BarryBozec, LaurentMiller, Francis J.Bradley, Elyse CornettMillet, ÓscarBranca, Jacopo Junio ValerioMilton, NathanielBrand, SergeMimica-Dukic, NedaBrash, AlanMinnelli, CristinaBriedis, VitalisMioc, MariusBrooke, GregMircioiu, ConstantinBrougham, DermotMirza, SabiruddinBrozovic, AnamariaMishra, NigamBrulíková, LucieMistiaen, WilhelmBruzzone, SantinaMitulovic, GoranBrzeziński, KrzysztofMiyamoto, HirotakaBuchet, ReneMizumura, KazueBucolo, ClaudioMlynarczyk, DariuszBudai-Szűcs, MáriaMobbili, GiovannaBudzevich, MikalaiMoccia, StefaniaBugarin, AlejandroMojzych, MariuszBungau, Simona GabrielaMoldoveanu, Serban C.Buonaguro, LuigiMolino, PaulBurak, M.FurkanMollace, VincenzoBurashnikov, AlexanderMoncharmont, BrunoBurchacka, EwaMondal, GoutamBurger, Michael CMondanelli, GiadaBurghardt, KyleMontalbàn, Mercedes G.Butnariu, MonicaMonteiro, CarlosCadelis, Melissa M.Moon, Jong-SeokCaffrey, ConorMorais, Tânia S.Cai, YingyunMorak-Młodawska, BeataCai, ZhengxinMoreau, RegisCajal, YolandaMoreira, Vânia M.Calina, DanielaMorel, BertrandCalvo, PilarMoretti, MassimoCambray, SerafíMorgan, Ethan L.Campisi, GiuseppinaMorgat, ClémentCaneiras, CátiaMorikawa, SatoruCapasso, DomenicaMorikawa, ToshioCapasso, RaffaeleMorris, Stephen A.Capperucci, AntonellaMorrow, Janet R.Cappuccilli, MariaMorzycki, Jacek W.Capuano, AnnalisaMoschini, RobertaCaputo, GregoryMot, AugustinCaramella, Carla MarcellaMottaghipisheh, JavedCardenas Zuniga, RobertoMoyá, María LuisaCardullo, NunzioMucha, IgorCarella, Angelo MMueller, CristinaCaron, LeslieMuenzberg, MichaelCarotenuto, MarcoMuhammad, NaoshadCarrà, GiovannaMuniyan, SakthivelCarradori, SimoneMurphy, Daniel J.Carrella, SabrinaMusteata, MihaiCarrier, PaulMusumeci, DomenicaCarrizzo, AlbinoMusumeci, RosarioCarter, Lukas M.Myung, WoojaeCarullo, GabrieleNabissi, MassimoCasini, GiambertoNagy, MiklósCastanho, MiguelNajib, Jadwiga S.Casteleijn, MarcoNajlah, MohammadCastoria, GabriellaNaka, HiroakiCastorina, AlessandroNakamaru-Ogiso, EikoCastresana, Javier S.Nakano, KenjiCastro, HelenaNakata, NorioCasu, CarlaNakhjavani, MaryamCaviglia, Gian PaoloNamgaladze, DmitryCecchinato, ValentinaNanavati, CharviCeci, FrancescoNarcisa, MandrasCerchione, ClaudioNatsumeda, ManabuCerdán, María-EsperanzaNavon, GilCerofolini, LindaNehela, YasserCerón-Carrasco, José PedroNekhai, SergeiCerqueira, FatimaNeophytou, Christiana M.Cerrito, Maria GraziaNg, Ho LeungČesla, PetrNg, Roy Chun-LaamCespi, MarcoNiculae, DanaCetinel, SibelNieoczym, DorotaChakraborty, JoydeepNiidome, TakuroChan, Ben C. L.Nikolakakis, IoannisChan, Koon HoNikolay, RainerChang, Ching-yaoNikolett, KállaiChang, Ling-ChuNisbet, Rebecca M.Charykov, Nikolay A.Nishi, KosukeChen, Chuan-LinNitulescu, MihaiChen, Jen-TsungNiwano, YoshimiChen, Qiao-HongNoguchi, YoshihiroChen, XiaoxinNoguera, Nélida InésChen, XinNomani, AlirezaChen, Yih-YuanNoren Hooten, NicoleChen, Yu-KuoNoszczyk, Bartłomiej HenrykChen, Yun-JuNotarbartolo, MonicaCheng, HaiziNowicka, PaulinaCheng, JosephNtanasis-Stathopoulos, IoannisCheng, Luisa W.Nuţǎ, Diana CameliaCheng, Yuan-BinNwabudike, Lawrence ChukwudiCheon, Yong-PilNylen, CarolinaCheong, Yuen-KiO’Bryan, John P.Cheung, AnthonyO’Connor, MichaelChhabra, GaganObata, YasukoChiarini, AnnaObi, ShuntaroChiesa, EnricaObreza, AlešChihuri, StanfordOgawa, KazumaChiriac, AuricaOh, ByungchulChisholm, John D.Oh, Eun-TaexChitgupi, UpendraOh, Jae-WookChmielewska, AleksandraOhlmann, Andreas V.Chobot, VladimírOhoka, NobumichiChoi, JunyongOkuda-Ashitaka, EmikoCholewinski, GrzegorzOlar, RodicaChoquesillo-Lazarte, DuaneOlaru, Octavian TudorelChoromanska, AnnaOliver, Allen G.Christoforides, EliasOniga, IlioaraChubarov, AlexeyOniga, Smaranda DafinaChueh, Pin JuOniszczuk, AnnaChung, Seung WooOnnis, ValentinaCiani, YariOnuki, YoshinoriCiccone, LidiaOpaliński, ŁukaszCicenas, JonasOramas-Royo, SandraCicero, ArrigoOrlando, TomasCichero, ElenaOrłowski, MarekCiesla, LukasOrsi, MarioCioce, MarioOrtega, Miguel A.Ciofalo, MaurizioOrtega-Gutierrez, SilviaCipakova, IngridOrtiz-Masiá, DoloresCiriaco, FulvioOtsuki, TakemiCirri, DamianoOzen, MehmetCiura, KrzesimirPaduch, RomanCivita, ProsperoPagano, CinziaČizmić, MirtaPakhomova, SvetlanaClanchy, FelixPalioura, SotiriaClement, YuriPalla, FrancoCobley, JamesPalmerini, MariaCochet, ClaudePalmieri, ErikaColl, RebeccaPalomo, ValleCollina, SimonaPaloncýová, MarkétaColotta, VittoriaPalugan, LucaColpo, GabrielaPan, JingColuccia, MauroPanwar, PriyankaComai, GiorgiaPanzarini, ElisaComfort, Kristen K.Papakyriakou, AthanasiosConcellon, AlbertoPapp, BélaConciatori, FabianaParang, KeykavousConejos-Sanchez, InmaculadaParenti, CarmelaConverti, AttilioParis, JuanCopmans, DaniëllePark, Dong SunCorbett, YolandaPark, Eun JeongCordeiro, Rosemeyre AmaralPark, Jong KookCornelison, Christopher T.Parsons, RichardCornett, ClausPashov, AnastasCorsi, LorenzoPasini, LuigiCortese, KatiaPašková, LudmilaCosma, PinalysaPasquale, Elena BiancaCotsoglou, ChristianPatini, RomeoCoudert, Amélie E.Patiño, José Luis CopaCoupland, Lucy A.Patra, Hirak K.Couture, RéjeanPatruno, AntoniaCoy-Barrera, EricssonPatterson, JenniferCozza, GiorgioPatyra, EwelinaCristiano, Maria De LurdesPaulo, AntónioCrocetti, LetiziaPavic, ValentinaCruz, GonzaloPavlovic, MarkoCsámpai, AntalPawlik, AndrzejCsiszar, AgnesPawlikowska-Pawlęga, BożenaCsuk, RenéPeana, Alessandra T.Cui, QingbinPecho-Vrieseling, ElineCui, YanxiangPeifer, ChristianCurti, DanielaPeitsidis, PanagiotisCutone, AntimoPellico, JuanCyr, ClaudePeluso, PaolaCzaplicki, SylwesterPeña Fernández, María ÁngelesCzernel, GrzegorzPendyala, Gurudutt N.Czerwińska, MonikaPeppelenbosch, M.P.D’Agnano, IgeaPerdih, FrancD’Errico, StefanoPereira, Nelson A. M.Da Silva Candal, AndrésPereira, SóniaDal Ben, MatteoPereira-Leite, CatarinaDandawate, PrasadPérez Pérez, MaríaDanesi, RomanoPérez, SalvadorDanko, MartinPerinelli, Diego RomanoDansette, Patrick M.Perioli, LuanaDardonville, ChristophePero, RaffaelaDarenskaya, M. A.Perrino, Enrico VitoDario, BrunettiPeruzynska, MagdalenaDas, SiddharthaPešić, MilicaDaśko, MateuszPethő, ZoltánDavies, AlexanderPetrak, JiriDe Araujo, ElvinPetrou, ChristosDe Berardis, DomenicoPetrova, GuenkaDe Bock, MarijkePhan, Chin-SoonDe Caro, VivianaPiciu, DoinaDe Carvalho, Litia AlvesPierreeiche, OlivierDe Filippis, BarbaraPilato, FabioDe Martin, RainerPilkington, LisaDe Masi, LuigiPilli, NageswaraDe Pasquale, ClaudioPilmane, MāraDe Simone, AngelaPilvankar, MinuDe Vita, DanielaPinela, JoséDebinski, WaldemarPiper, BrianDefant, AndreaPirman, TatjanaDel Rio, DanielePiróg, Katarzyna A.Delage, JudithPisanu, ClaudiaDembinski, RomanPishchalnikov, Roman Y.Demopoulos, Vassilis J.Pisklak, Dariusz MaciejDémuth, BalázsPitucha, MonikaDenevault, CarolinePiva, RobertoDenkova, AntoniaPlanas, MartaDepciuch, JoannaPlastina, PierluigiDesai, AmritaPlazinski, WojciechDettlaff, KatarzynaPoddelsky, AndreyDevroye, CélinePodlewska, SabinaDhakal, DipeshPoggi, AlessandroDi Cesare Mannelli, LorenzoPojo, MartaDi Giacomo, SilviaPolano, MaurizioDi Micco, SimonePolini, BeatriceDi Natale, ConcettaPolishchuk, PavelDi Paolo, AntonelloPonce-Vargas, MiguelDi Pinto, AnnaPonnandy, PrabhuDiakopoulos, NinaPontrelli, PaolaDias Soeiro Cordeiro, Maria NataliaPop, CristinaDimitrakopoulos, Foteinos-IoannisPopa, Mircea IoanDingley, Andrew J.Popat, AmiraliDinischiotu, AncaPopa-Wagner, AurelDoda, Sai ReddyPope, ChadDoligalska, MariaPopeijus, Herman E.Domínguez Álvarez, EnriquePopescu, Mihaela R.Domoki, FerencPorcheddu, AndreaDonato, LuigiPotaczek, Daniel P.Dorfman, RuslanPouliot, MarcDoris, EricPourová, JanaDorotea, Muck-SelerPoutou, JoannaDossi, CarloPoveda Larrosa, Jose AntonioDouroumis, DennisPoznański, JarosławDrabowicz, JozefPramod, KannisseryDragos, HorvathPrange, ReinhildDrici, Milou-DanielPrevet, NellaDrosten, MatthiasPriego, Eva-MariaDroździk, MarekPrieto, Ana Maria SahagunDrummer, OlafPrima, Giulia DiDuan, WeiPrimavera, RositaDuarte, NoeliaPrimožič, InesDuatti, AdrianoPrzybył, Jarosław L.Dubiel, WolfgangPsurski, MateuszDubrana, KarinePulvirenti, AlfredoDuburs, GunarsQadura, MohammadDuda, Marcel MateiQin, HuaDudhipala, NarendarQuadri, Syed A.Dufrasne, FrancoisQuaini, FedericoDugar, Rohit P.Queiroga, FelisbinaDuong, Van TuQuiliano, MiguelDurand, NishaQuiroga, Adoracion GomezDuranti, AndreaRacz, IldikoDurazzo, AlessandraRadaram, BhaskerDurgo, KsenijaRadchenko, Eugene V.Dutta, PranabanandaRafaniello, ConcettaDziedzicka-Wasylewska, MartaRahimian, JosephDzięgiel, PiotrRahman, SafikurEadon, Michael T.Rajakumar, NagalingamEar, JasonRakic, JelenaEbrahim, WeaamRamadori, GiulianoEdhi, Muhammad MuzzammilRamos, CarmenEedara, Basanth BabuRamsbeck, DanielElansary, Hosam O.Ran, YanEldeeb, SamiRana, JyotiEleftherianos, IoannisRanzato, EliaEleonora, RussoRapposelli, SimonaEl-Moghazy, AhmedRasapalli, SivappaEl-Sayed, Naglaa SalemRascher, WolfgangEl-Seedi, HeshamRashid, Mohammad HarunEmadi, AshkanRastija, VesnaEmri, EszterRastrelli, LucaEndre Károly, KristófRauch, BernhardEppard, ElisabethRavegnini, GloriaErsilia, AlexaRebollo-Hernanz, MiguelEscribano, JulioReid, Christopher W.Eskin, MichaelReid, St PatrickEspinosa-Diez, CristinaReis, CatarinaEstenoz, DianaReiss, Lucinda V.Estévez, JorgeRejinold, SanojEufemi, MargheritaRen, ZhenhuaEvans, MalkanthiRenukuntla, JwalaEvans, MichaelResendiz, MarinoFabiani, RobertoRey Rios, AnaFailla, Cristina M.Reynisson, JóhannesFalzone, LucaRhaleb, Nour-EddineFanale, DanieleRibaudo, GiovanniFang, XingRiccardi, ClaudiaFasler, ElizavetaRicciutelli, MassimoFatouros, Dimitrios G.Richardson, Simon C. W.Faustino, RandolphRiera, AntoniFebbraio, FerdinandoRigano, LuigiFede, CaterinaRigo, RiccardoFedele, MonicaRipandelli, GuidoFeher, GergelyRishi, GautamFeier, BogdanRizzo, GianlucaFelczak, AleksandraRoberto, MichelaFeliciano, JoanaRobitzsch, AlexanderFeng, Hua-JunRocca, RobertaFeraco, PaolaRocha, JoãoFerrante, ClaudioRochani, AnkitFerraz, RicardoRodrigues, António SebastiãoFerri, NicolaRodrigues, MárcioFerrini, SilvanoRodríguez, Armando AlexeiFestuccia, ClaudioRodríguez-Lozano, Francisco JavierFik-Jaskółka, Marta A.Rodriguez-Rubio, LorenaFinke, Jan HenrikRodriquez, ManuelaFior, RitaRohacs, TiborFischer-Fodor, EvaRolo, JoanaFitz, Nicholas F.Romeiro, NelilmaFlak, Jonathan N.Ronca, RobertoFleisher-Berkovich, SigalRosenström, UlrikaFliefel, Riham M.Roszczenko-Jasinska, PaulaFlorens, NansRothan, HussinFoerster, Kathrin I.Roy, Saktimayee MitraFogacci, FedericaRudd, SeanFontés, MichelRuiz, MarioForzato, CristinaRund, DeborahFracchiolla, Nicola StefanoRusso, DomenicoFraguas-Sánchez, Ana IsabelRusso, EthanFranco, Carlos M.Russo, Mario VincenzoFrański, RafałRutigliano, GraziaFranzoni, GiuliaRyan, MichaelFrasca, LoredanaRybka, JakubFrazzi, RaffaeleRyszawy, DamianFresco-Cala, Beatriz MaríaRyu, Ji HyunFrezza, ClaudioSaaby, LasseFromm, BastianSaclier, MarielleFrosini, MariaSaczewski, JarosławFuentes, EduardoSafa, Ahmad R.Fujimori, KoSagheddu, ClaudiaFujita, MasakiSaha, BiswajitFunaki, MakotoSahni, Sanjeev K.Füredi, AndrásSahu, BhubananandaFurney, Simon J.Saisho, YoshifumiFuruta, SaoriSaitoh, AkiyoshiFusco, SalvatoreSakurada, YoichiGackowski, DanielSalas, José BlancoGaertner, Florian C.Saldanha, SabitaGajewicz-Skretna, AgnieszkaSaleh-E-In, Md. MoshfekusGajos-Michniewicz, AnnaSalvati, AnnamariaGál, PeterSalvati, EricaGalanakis, Charis M.Samsonov, SergeyGaletti, MariclaSánchez Navarro, AmparoGáll, ZsoltSankaranarayanan, Nehru VijiGallo, CarmelaSansone, AnnaGan, RenyouSantarelli, AndreaGan, SamuelSantini, CarloGangaraju, RajashekharSantos-Filho, Osvaldo AndradeGao, JieSantucci, AnnalisaGao, JinSantulli, GaetanoGarbers, ChristophSapio, LuigiGarcía Honduvilla, Natalio AntonioSardo, AngelaGarcia, Brandon L.Sasmal, AniruddhaGarcia-Doval, CarmelaSasso, Ferdinando CarloGarcia-Gimenez, Maria DoloresSato, HideyukiGarcia-Pardo, JavierScacco, SalvatoreGarcia-Sosa, AlfonsoSchapowal, AndreasGarrosa, ManuelSchenone, SilviaGassman, NatalieScheunemann, MatthiasGaudencio, SusanaSchindowski, KatharinaGe, RuowenSchinnerl, JohannGęgotek, AgnieszkaSchlüter, Klaus-DieterGener, PetraSchmid, AndreasGentile, CarlaSchmidt-Wolf, Ingo Gh H.Gentile, FabrizioSchmitt, SébastienGeorgiev, VasilScholz, MartinGerogiorgis, DimitriosSchramm, AlexanderGerrard, GarethSchröder, LeifGetz, GodfreySchulze-Tanzil, GundulaGhersi, GiulioSchweizer, FrankGhiciuc, Cristina MihaelaSciamanna, GiuseppeGhori, Muhammad UsmanScorziello, AntonellaGiamperi, LauraSegale, LorenaGianferrara, TeresaSegatto, MarcoGikas, EvagelosSehlin, DagGill, Pritmohinder S.Selin, VictorGiménez Bastida, Juan AntonioSelivanova, Svetlana V.Gitlin-Domagalska, AgataSellars, JonathanGiunchedi, PaoloSeo, SungbaekGlassman, Patrick M.Serwer, PhilipGnat, SebastianSethi, GautamGobeil, Fernand Jr.Sgarlata, CarmeloGoda, KatalinShah, Dhaval K.Godin, BianaShah, Furqan A.Godlewska, MarlenaShaik, Mohammed RafiGohda, EiichiShakya, AkhileshGolay, JoseeShariare, MohammadGolestaneh, LadanSharma, AbhisheakGolla, UpendarSharma, RitinGoltsov, AlexeySharova, NataliaGómez-Muñoz, María TeresaSherpa, Rinzhin TsheringGómez-Rincón, CarlotaShi, JianGonkowski, SławomirShi, ZengqianGonzalez, Miguel ToroShi, ZhiGonzález-Navarro, HerminiaShidal, ChrisGoodier, Martin R.Shigdar, SarahGoodwani, SunilShim, Jae-HyuckGorbacheva, LiubovShin, Dong HoonGori, TommasoShin, Meong CheolGorodetsky, RaphaelShinoda, KeiGoto, KenichiShirato, KenGoulle, Jean-PierreShu, XinhuaGovan, JosephShuichi, TakayamaGranados-Principal, Sergio M.Shukla, KirtikarGregory, StevenSiczek, KrzysztofGriesbeck, Axel G.SIedlewicz, GrzegorzGrimaldi, PaolaSiejka, AgnieszkaGrimm, Marcus O. W.Sigala, SandraGrimm, Wolf-DieterSignore, GiovanniGrubić Kezele, TanjaSikora, JoannaGrünberg, JürgenSikorski, Aleksander F.Grzeskowiak, BartoszSilva, AndreGubernator, JerzySilvestri, GiovanninoGuix, Francesc XavierSimone, ElenaGunaje, JayaramaSimpson, David A. C.Guo, XinSingh, AnandGurrapu, SreeharshaSingh, SeemaHagag, AhmedSipos, EmeseHagerman, RandiSiristatidis, CharalamposHalatsch, Marc-EricSistla, SrinivasHalwachs, BettinaSitarek, PrzemysławHamashima, YoshitakaSivaev, Igor B.Hamed, Ashraf Nageeb ElsayedSivakumar, SushamaHamilton, GerhardSkenderidis, ProdromosHampp, NorbertSkořepová, EliškaHan, Sun-YoungSkrypnik, KatarzynaHan, YuyanSkupin-Mrugalska, PaulinaHanaoka, HirofumiSlama, James T.Hanna, Ramy M.Śliwa, PawełHano, ChristopheSliwinski, TomaszHao, JinsongSloan, Alastair J.Haro, IsabelSłoczyńska, KarolinaHartman, MariuszSlowinska, KatarzynaHashemolhosseini, SaidSmiddy, William EHealey, GarethSnowden, TimothyHeilbronner, UrsSobeh, MansourHeinbockel, ThomasSobhy, HaithamHeitmar, RebekkaSocała, KatarzynaHellinger, RolandSokolová, RomanaHennkens, Heather M.Solomonov, InnaHennrich, UteSolovieva, SvetlanaHerbet, MariolaSoltész, BeátaHernández, Sara B.Song, Im-SookHernandez-Olmos, VictorSorsa, TimoHerrera, FedericoSoural, MiroslavHilhorst, MarcSousa, Sérgio F.Hillard, Cecilia J.Sova, MatejHindocha, ChandniSpanakis, MariosHinkula, JormaSparkenbaugh, Erica M.Hinrichs, Wouter L. J.Spartalis, MichaelHinton, CimonaSpirina, Liudmila V.Hirabayashi, JunSpiwok, VojtechHirayama, FumitoshiSreerama, SubramanyaHodges, H. CourtneySrivastava, Swayam PrakashHofman, TadeuszSt. Louis, IrinaHohenegger, MartinStacewicz-Sapuntzakis, MariaHolas, OndřejStadlbauer, SvenHolbech Jensen, Benjamin AnderschouStaedler, DavideHolsinger, DamainStana, Anca - DanielaHolstein, SarahStanciu, Gabriela-DumitritaHolt, AndrewStankovic, MilanHoneywell, Richard J.Štarha, PavelHoogland, DominiqueStark, HolgerHosui, AtsushiStavraka, CharaHou, HarveyStawny, MaciejHrast, MartinaSteardo, LucaHryniewicka, AgnieszkaStec, JozefHsia, Shih-MinStefanidou, Maria E.Hsiao, Chung-DerStella, Giulia M.Hsieh, Hsi-LungStepensky, DavidHsieh, Jui-HuaStephens, GaryHsieh, Shu-ChenStrekowski, LucjanHsieh, Wen-TsongStruga, MartaHsieh, Yi-HsienSturlese, MattiaHsin, Kun-YiSu, Chinh Tran-ToHsu, Hao-JenSu, DanHsu, Kuo-ShengSubrizi, AstridHsu, Yu-WenSuffritti, ChiaraHu, Dong GuiSugimura, HaruhikoHuang, Chien-ChungSui, YongjunHuang, Jiann-JyhSukocheva, OlgaHuang, Paul Li-HaoSun, Changquan CalvinHudson, NatalieSupuran, ClaudiuHughes, LouiseSut, StefaniaHung, Chin-ChuanSuzuki, MasaakiHussain, Ali A.Suzuki, ToyofumiHussain, HidayatSuzuki, YukiHydbring, PerSvircev, Antonet M.Hytti, MariaSvoboda, KurtIannolo, GioacchinŚwiątek, ŁukaszIasevoli, FeliceŚwiderek, KatarzynaIeguchi, KatsuakiSzallasi, ArpadIelciu, IrinaSzandruk, MartaIglesias-Osma, Maria CarmenSzeliga, MonikaIgotti, MariaSzewczuk, Myron R.Imai, JunSzewczyk-Golec, KarolinaImran, SaimaSzlęk, JakubIndra, RadekSznitowska, MałgorzataIndrakumar, SowmyaSztanke, MałgorzataIngle, SuzanneSzulczyk, DanielIngram-Smith, CherylSzyling, JakubIonescu, Mihaela IleanaTabachnik Schor, Nina F.Ionita, PetreTada, RuiIshida, TsuneoTakashi, YuichiIshii, KenichiroTakeda, ShigekiIshikawa, TomohiroTakegahara, NorikoIssa, SamarTakemoto, JonItarte, EmilioTalukder, PoulamiIvanov, Yuri D.Tambaro, SimoneIvanova, SvetlanaTamkovich, SvetlanaIzadyar, AnahitaTamura, RuiJ Edagwa, BensonTanabe, HirokiJakobusic Brala, CvijetaTanaka, HidenoriJanagam, Dileep ReddyTanaka, IchidaiJaneczko, MonikaTanase, CorneliuJanowski, MiroslawTang, JingJaśkowska, JolantaTao, YunwenJaworek, Andrzej KazimierzTarozzi, AndreaJayant, RahulTasso, BrunoJayaraman, SundararajanTavolari, SimonaJean, LudovicTchetina, Elena V.Jensen, Knud JørgenTcholakova, SlavkaJensen, Svend BorupTejera, EduardoJeong, Jae MinTerreehorst, IngridJi, BinTessem, Jeffery S.Jia, KailiangTesta, UgoJia, ShangTezera, Liku BekeleJiandong, HuangThangavel, ChellappagounderJiang, Jhih-HangThevkar, PrashanthJibran, RubinaTietjen, IanJijie, RoxanaTietze, RainerJoers, ValerieTing, Jeffrey M.John Jervis, PeterTinti, FrancescaJohnson, Elizabeth O.Tiperciuc, BrindusaJohnston, Graham A.R.Tircso, GyulaJones, BenTiso, NatasciaJordan, SueToennes, Stefan W.Jorge Guiomar, AntónioTognolini, MassimilianoJorgenson, MatthewTombelli, SaraJunichi, KitanakaTomek, PetrJunquera, LuisTomoda, HiroshiJuszczak, LesławToro, Mario DamianoJuvik, John A.Torrado, Juan J.Kaarniranta, KaiTorre, Maria LuisaKabała-Dzik, AgataTorregrosa-Crespo, JavierKabir, Mohammad FaujulTorres Sangiao, EvaKaburagi, TomokoTorrijos, Trinidad VelascoKaczyńska, KatarzynaTortorella, PaoloKadam, AvinashTreglia, GiorgioKaddour, HusseinTsai, RongkungKadela-Tomanek, MonikaTsai, Ying-ChiehKaesler, SusanneTsakovska, IvankaKagawa, TatehiroTseng, Chih-HuaKairys, VisvaldasTseng, Kuang-WenKaiser, Thomas M.Tsinopoulos, IoannisKakkassery, VinodhTsioutis, ConstantinosKalas, WojciechTsuboi, HirohitoKalinina, Elena V.Tsuchiya, HiroyukiKalisz, StanisławTsugawa, HitoshiKalogiouri, NatasaTsytsarev, VassiliyKamolz, Lars P.Tuccinardi, TizianoKanaujia, ParijatTundis, RosaKANEDA, TakeharuTungmunnithum, DuangjaiKang, Jeong-HunTural, ÜmitKantminienė, KristinaTurk, FlorianKapusta, IreneuszTutone, MarcoKardos, JuliannaTyszka-Czochara, MalgorzataKarolewicz, BożenaUeno, MasakiKarpinski, Tomasz M.Umemura, NaokiKashman, YoelUmerska, AnitaKaskel, Frederick J.Unchwaniwala, NuruddinKaškonienė, VilmaUrbanczyk-Lipkowska, ZofiaKasprzak, ArturUrban-Wojciuk, ZuzannaKattner, LarsUribe, Kepa B.Katyal, PriyaUsai, DonatellaKaur, TanpreetUsai, MariannaKawakami, SusumuUsui, YoshihikoKawanami, DaijiUziel, OritKawczak, PiotrValentová, KateřinaKazakova, Oxana B.Valero, Marta SofíaKejík, ZdeněkVallet, SoniaKeller, AdrianVan Aerschot, ArthurKesharwani, SiddharthVan Balkom, Bas W. M.Khairullina, VeronikaVan Dyke, MichaelKhajehei, ForoughVan Laar, TriciaKhambu, BilonVandooren, JenniferKhammanivong, AliVarano, FlaviaKhanra, NandishVarela-Ramirez, ArmandoKholmukhamedov, AndalebVassanelli, AuroraKhomutov, Alex R.Vasudevan, SreelakshmiKhurana, NamrataVeeramani, SureshKhurana, VarunVek, ViljemKiesewetter, Dale O.Velasco-Torrijos, TrinidadKilari, SreenivasuluVelez, ZéliaKim, BongleeVelikyan, IrinaKim, HoonVemula, HarikaKim, JoungmokVentura, Cinzia AnnaKim, NamkwonVentura, NatasciaKim, Pyung-HwanVerhoeven, AdrieKim, Su YoungVerma, Shiv PrakashKim, Woo-YangVerstegen, Monique M. A.Kinzhalov, Mikhail A.Vibhute, Sandip M.Kishimoto, DaigaViggiano, AndreaKishore, KamalVilela, ManuelKissi, EricVillaverde, GonzaloKitano, YoshikazuVilloutreix, BrunoKlahn, PhilippVitetta, LuisKleckner, Amber S.Vlachou, MarilenaKleczkowska, PatrycjaVlainić, JosipaKlink, MagdalenaVlase, TitusKlotz, ChristianVoliani, ValerioKluczyk, AlicjaWagner, Kay-DietrichKocot-Kępska, MagdalenaWähälä, KristiinaKoh, Yong QinWahli, WalterKohler, James J.Walker, SteveKoike, TomoyukiWalter, Fruzsina R.Kole, ChristoWalter, UlrichKolesińka, BeataWang, Chao-MinKoltai, HinanitWang, ChenguangKonar, SumitWang, GaojiKondo, HiromuWang, GuangshunKonstantinidis, Theocharis G.Wang, Hui-ChunKontogiannidou, EleniWang, KimberleyKopaczyńska, MartaWang, LipingKopustinskiene, Dalia M.Wang, LirongKormos, AtillaWang, MingmingKostka, LiborWang, Mong-HengKostoulias, XeniaWang, RuiyongKourkoutas, IoannisWang, ShuminKouza, MaksimWang, Yeng-TsengKovacs, RenatoWang, YingKovatcheva-Datchary, PetiaWang, ZhijunKowalska, JoannaWarnken, ZacharyKowalski, RadosławWarren, J. DavidKoyioni, MariaWeaver, JolantaKozakiewicz, MariuszWeiss, JohannaKozempel, JánWells, Timothy N. CKozlov, SergeyWen, ZhiweiKrakowiak, AgnieszkaWeng, Yung-JinKrammer, Eva-MariaWenzel, MichaelaKrause, FrankWhite, C. MichaelKrawczynski, KamilWhite, SimonKrijt, JanWhitlock, ChristineKrishna, GokulWieczorek, PiotrKról, EwelinaWilkins, HeatherKropp, MartinaWilkinson, JennyKrukiewicz, KatarzynaWilliams, AlfredKrummenacher, ClaudeWinckler, ThomasKrzyściak, WirginiaWinter, Arthur H.Krzystanek, MarekWnuk, AgnieszkaKubica, PawełWnuk, MaciejKubinova, SarkaWójciak, MagdalenaKucinska, MalgorzataWolf, StevenKucuk, IsrafilWozniak, KatarzynaKulkarni, PriyankaWu, Sheng-NanKumar, ManishWujec, MonikaKumari, HarshitaXavier, Nuno M.Kumari, NeelamXiao, BoqiKume, KensukeXie, WankunKuminek, GislaineXu, ChuanmingKunii, YasutoXue, GuangaiKurczab, RafałYadav, VinitaKuryk, LukaszYallapu, Murali MohanKutner, AndrzejYamashita, ShinjiKwiatkowski, PawelYan, ZiyingKwon, SunohYang, ChunhuaKwon, Tae-HyukYang, Hsiao-YuKwon, Young M.Yang, JialeLa Motta, ConcettinaYang, Shun-FaLa Rosa, PiergiorgioYang, SiyoungLabudda, MateuszYang, YannanLach, Michał StefanYang, YongyongŁączkowski, Krzysztof Z.Yang, ZhihongLaher, IsmailYaremenko, Ivan A.Lai, AlexanderYarmolinsky, LudmilaLaity, Peter RYaron, Jordan R.Lamari, Fotini N.Yasuda, KazukiLambert, JoergYayoi, KawanoLambruschini, ChiaraYi, MyunggiLamouroux, EmmanuelYonemochi, EtsuoLamponi, StefaniaYoo, Hah YoungLan, LanYousefi, Behrooz H.Laneri, SoniaYu, ZhanyangLapucci, AndreaYuan, Hanna S.Larson-Prior, Linda J.Yurchenko, VyacheslavLasoń, WładysławZaiss, DietmarLauretani, FulvioZaitsev, AlekseyLazarowski, AlbertoZampogna, AlessandroLazzarato, LorettaZavan, BarbaraLe Borgne, MarcZawada, KatarzynaLebeau, LucŻelaszczyk, DorotaLedreux, AurélieZhang, ChunchaoLee, Chang-minZhang, KeqiangLee, Che-HsinZhang, LiangrenLee, Chia-HungZhang, LiqiangLee, Chia-HwaZhang, MingLee, Ching-KuoZhang, XuexinLee, ChoonghoZhang, YanlingLee, JinZhao, LinboLee, Meng-shiouZhao, MeixiaLee, MinaZhao, XiaoaiLee, SeungHwanZhao, YuLegrand, François-XavierZheng, Yun-WenLehman, John M.Zheng, ZhongLehmann, Lorenz H.Zielinska, AleksandraLei, WeiZiemssen, FockeLeiro, VictoriaZloh, MireLeisegang, Matthias S.Zmudzki, PawelLemli, BeataZobi, FabioLenci, ElenaŻołek, TeresaLenon, GeorgeŻołnowska, BeataLeon, AntonioZorc, BrankaLeone, MarilisaZou, WeiLeone, SheilaZucchelli, MircoLepage, MartinZuercher, BillLescure, AlainZustiak, Silviya Petrova

